# The Gray Area of Freezing of Gait Annotation: A Guideline and Open‐Source Practical Tool

**DOI:** 10.1002/mdc3.13556

**Published:** 2022-09-18

**Authors:** Helena Cockx, Emilie Klaver, Marleen Tjepkema‐Cloostermans, Richard van Wezel, Jorik Nonnekes

**Affiliations:** ^1^ Department of Biophysics Radboud University, Donders Institute for Brain, Cognition and Behaviour Nijmegen The Netherlands; ^2^ Department of Neurology and Clinical Neurophysiology Medical Spectrum Twente Enschede The Netherlands; ^3^ Clinical Neurophysiology group University of Twente Enschede The Netherlands; ^4^ Department of Biomedical Signals and Systems University of Twente Enschede The Netherlands; ^5^ Department of Rehabilitation; Centre of Expertise for Parkinson & Movement Disorders Radboud University Medical Centre; Donders Institute for Brain, Cognition and Behaviour Nijmegen The Netherlands; ^6^ Department of Rehabilitation Sint Maartenskliniek Nijmegen The Netherlands

**Keywords:** freezing of gait, video annotations, standardization

## Abstract

**Background:**

Freezing of gait, a disabling episodic symptom, is difficult to assess as the exact begin‐ and endpoint of an episode is not easy to specify. This hampers scientific and clinical progress. The current golden standard is video annotation by two independent raters. However, the comparison of the two ratings gives rise to non‐overlapping, gray areas.

**Objective:**

To provide a guideline for dealing with these gray areas.

**Methods/Results:**

We propose a standardized procedure for handling the gray areas based on two parameters, the tolerance and correction parameter. Furthermore, we recommend the use of positive agreement, negative agreement, and prevalence index to report interrater agreement instead of the commonly used intraclass correlation coefficient or Cohen's kappa. This theoretical guideline was implemented in an open‐source practical tool, *FOGtool* (https://github.com/helenacockx/FOGtool).

**Conclusion:**

This paper aims to contribute to the standardization of freezing of gait assessment, thereby improving data sharing procedures and replicability of study results*.*

Freezing of gait (FOG) in Parkinson's disease is often considered a well‐defined phenomenon, namely “a brief, episodic absence or marked reduction of forward progression of the feet despite the intention to walk.”^1^ However, in reality, the distinction between freezing and a person's regular gait pattern is not always an easy call to make (Fig. 1).^1^ The exact beginning and endpoint of a FOG episode is usually difficult to define, as FOG might evolve from a gradually worsening gait pattern, and it is not always clear whether “normal” gait is restored between two episodes of FOG.^2^, ^3^ These difficulties were reflected in a study comparing video annotations from 10 experienced raters from four different centers: they found a lower interrater agreement for the number of FOG episodes than for the percentage time frozen (intraclass correlation coefficient of 0.63 and 0.73, respectively), suggesting that some experts annotated one long episode whereas others annotated this as multiple short freezing epochs.^4^ Furthermore, the overall relatively low agreement indicates that clinicians frequently disagree on classifying an episode as freezing or not.^5^

**FIG. 1:**
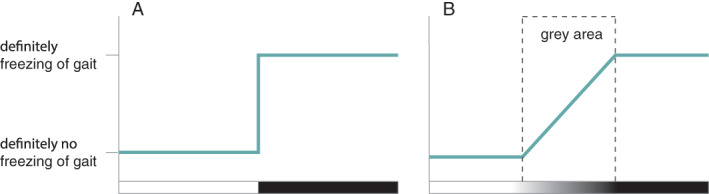
Freezing of gait is often considered a clearly defined phenomenon (A). However, there exists a large gray area between normal gait and freezing of gait (B), as freezing might evolve from a gradually worsening gait pattern, and it is not always clear whether normal gait is restored between two episodes of freezing.

The field is increasingly aware that the difficulties in FOG assessment are holding back our progress in unraveling the mechanisms underlying FOG and development of new treatment approaches. Indeed, a scientific panel recently emphasized that the development of standardized procedures and guidelines is crucial to make advances in our understanding of the underlying mechanisms of the symptom and eventually in the development of better treatments.^6^ Collaboration between centers and sharing of datasets is vital and further warrants coordinated and standardized procedures. Besides, the current lack of standards hampers the comparison of study results between centers, for example, to evaluate new FOG treatments.^7^

Currently, the golden standard for FOG assessment is based on video annotations.^4^, ^8^ Gilat recently proposed a good starting point to standardize this procedure by using the open‐source software ELAN.^9^ Of course, the ultimate goal is to develop a more objective detection algorithm based on wearable sensors (eg, accelerometers) which would be less time‐consuming and cumbersome than video annotations. However, the accuracy of the existing algorithms is currently insufficient and improvement of these requires large, video‐annotated datasets; hence stressing the growing need to share standardized data over centers.^10^, ^11^, ^12^

Given the high inter‐rater variability for FOG annotation, the current guideline states that at least two experts should annotate the videos. Although this was already recommended by several authors at the beginning of 2010,^4^, ^8^ only a minority of studies have been following this advice. We reviewed the annotation procedures of 74 studies included in a recent systematic review on wearable‐sensor‐based FOG detection and prediction algorithms.^10^ To our surprise, less than 20% of the studies reported that the annotations had been performed by two experts or more.

The comparison of the two annotations allows to identify episodes that definitely contain FOG (or not) when these are perfectly overlapping, but also gives rise to ambiguous, non‐overlapping, gray areas (Fig. 2, “overlap”). There is currently no consensus on how these gray areas should be handled. Previous methods, if described at all, ranged from averaging the observations to discussing the gray areas to get to a consensus.^4^, ^10^ Notwithstanding that we agree upon discussing episodes that are only identified by one of the raters, we believe that it is not necessary to discuss all minor differences in annotations at the start or end of an episode, as long as the procedures are described clearly.

**FIG. 2:**
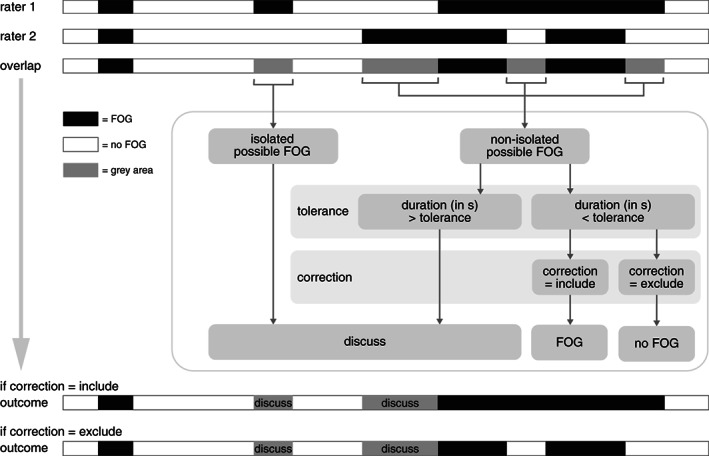
Explanation of the proposed procedure to combine the annotations of two raters. Completely overlapping annotations can definitely be considered as freezing (black) or no freezing (white), while the non‐overlapping areas (gray areas) are processed by the algorithm with the input of the “tolerance” and “correction” parameter. The remaining gray areas should be discussed with a third rater or until consensus is reached. See main text for a more detailed description.

In this paper, we propose a guideline and open‐source practical tool to handle the gray areas of freezing annotation by building upon the previous guideline of Gilat.^9^ In the first theoretical part, we propose a standardized procedure that allows researchers to report unambiguously how the annotations of two raters are combined, based on two chosen parameters, the tolerance, and the correction parameter. Furthermore, we discuss the advantages and disadvantages of some agreement and reliability parameters, including the intraclass correlation coefficient and Cohen's kappa. In the second part, we give a short overview of how our open‐source tool, *FOGtool*, implements the proposed framework.

## Methods and Results

### Theoretical Guideline

Fig. 2 summarizes the proposed guideline. First, the annotations of both raters are compared. Completely overlapping parts can definitely be considered as freezing episodes (black areas) or non‐freezing episodes (white areas), while the non‐overlapping parts (gray areas) are further processed and have three possible outcomes: they will either be (1) included as FOG, (2) excluded as FOG, or (3) be considered for discussion.

The gray areas are split into isolated possible FOG episodes and non‐isolated possible FOG episodes. An isolated possible FOG episode, is an episode that was only annotated by one of the raters and should always be discussed with a third rater or until consensus is reached. A non‐isolated possible FOG episode is a part that borders a definite FOG episode, either by preceding one, following one, or being enclosed by one. The outcome of the latter episode depends on two parameters: the tolerance and the correction parameter.

#### Tolerance

Video annotations will never overlap perfectly. For example, rater 1 might start annotating the same FOG episode half a second earlier than rater 2. Although there is no substantial disagreement between the raters for this episode, this yields a short gray area with no further need for discussion. However, when the annotations would differ by 3 s, there might be a substantial disagreement about the start of the episode. The distinction between a minor imprecision in video annotation and a substantial disagreement in FOG assessment can be set by the tolerance parameter. Although this parameter can be freely chosen by the researcher, our advice is to set it at 2 s, according to the standardized definition for the end time of a FOG episode (“the participant is able to perform at least two effective alternating steps”) which corresponds to 2 s approximately.^9^ Fig. 2 displays the procedure for a non‐isolated possible FOG episode: if the episode is longer than the pre‐set tolerance, this part should be discussed. However, if the episode is shorter than the tolerance, this part is included or excluded as FOG based on the correction parameter.

#### Correction

The decision to consider a short, non‐isolated possible FOG episode (i.e., a bordering gray area with a duration of <2 s) as freezing or not, might depend on the situation. On the one hand, some researchers might only be interested in epochs that certainly contain FOG, for instance in fundamental studies aiming to unravel the pathophysiological substrate underlying FOG. In this case, the correction is set to “exclude” and the gray area will not be considered as FOG. On the other hand, some researchers might want to include all potential FOG epochs, for instance when developing an algorithm to provide on‐demand cueing. In this case, the correction is set to “include” and the gray area will be incorporated by the bordering FOG. The two bars at the bottom of Fig. 2 show the two possible outcomes when the correction parameter is set to “include” or “exclude”, respectively.

#### Interrater Agreement

Regardless of the decision to include or exclude the gray areas, the initial overlap between the two raters gives an indication about the reliability of the achieved annotations. A variety of agreement and reliability parameters have been reported in previous studies, of which the intraclass correlation coefficient (ICC) and Cohen's kappa have been reported most often. However, we argue that these are not the ideal reliability and agreement parameters when it comes to FOG assessment, as the ICC does not reflect the exact overlap of FOG events, and the Cohen's kappa tends to underestimate the agreement when events are rare. Instead, we promote the use of positive agreement, negative agreement, and prevalence index. An overview of the definitions and limitations of the different agreement parameters are given in Table 1. Formulas are given in the supplementary material.

**TABLE 1:** Definitions and limitations of reliability and agreement parameters

	Intraclass correlation coefficient (ICC)	Cohen's kappa		Positive agreement	Negative agreement	Prevalence index
**General definition**	Reliability measure indicating the degree of correlation for a continuous metric over different raters.^13^	Agreement measure between two raters, taking into account the probability of agreement occurring by chance.^14^		Degree of agreement between two raters for the positive category, given the distribution of responses.^15^	Degree of agreement between two raters for the negative category, given the distribution of responses.^15^	Relative prevalence of the positive compared to the negative category.^16^
**Description with regards to FOG assessment**	Correlation between raters on their overall FOG score (e.g., % time frozen) over the assessed participants.	Exact overlap between the annotated FOG events between raters, taking into account the probability of guessing.		Probability measure for the overlapping FOG events (= black areas).	Probability measure for the overlapping no FOG periods (= white areas).	Overall prevalence of FOG events.
**Interpretation**	See Cohen's kappa	<0.00	Poor^17^	See Cohen's kappa	See Cohen's kappa	−1 no FOG
		0.00–0.20	Slight			+1 continuously FOG
		0.21–0.40	Fair			
		0.41–0.60	Moderate			
		0.61–0.80	Substantial			
		0.81–1.00	Almost perfect			
**Limitations**	Does not reflect the exact overlap of the FOG events.^13^	Kappa tends to underestimate the agreement when the events are rare.^14^, ^19^		All three metrics are needed to evaluate the agreement between raters.		
	There exist many different formulas to calculate the ICC, each with a slightly different purpose.^18^	When both raters annotate no FOG episodes for a certain participant, Cohen's kappa becomes undefined.^14^				
**Advice**	Limit the use of ICC to studies where the exact overlap between episodes is not important (e.g., evaluation of FOG treatments) and always report the used formula.	Consider reporting positive agreement, negative agreement, and prevalence index instead of Cohen's kappa.^15^, ^16^		Report all three metrics together		

*Note*: Formulas of the different agreement parameters are given in the supplementary material.

Abbreviations: FOG, freezing of gait; ICC, intraclass correlation coefficient.

### Practical Tool

To implement the above proposed theoretical framework, we developed the “*FOGtool*”, an easy‐to‐use, open‐source, MATLAB‐based practical tool. In summary, videos are first annotated by two raters as previously proposed by Gilat in the ELAN software, including characterizing the phenotype (i.e., trembling, shuffling, or akinesia) and trigger (e.g., FOG_Target, FOG_180_R, FOG_Doorway) of each FOG episode.^9^ The annotations are subsequently exported as tabular files which can be read in by our stand‐alone software. The FOGtool will then, based on the chosen tolerance and correction parameter, define the agreed epochs (FOG or no FOG) and the to‐be‐discussed gray areas. Furthermore, FOG episodes that were annotated by both raters, but were characterized by a different phenotype or trigger, are flagged by a “check_type” or “check_trigger,” respectively. The outcome is visualized (similar to Fig. 2) and exported as tabular files which can be reimported into ELAN. Hence, researchers can discuss the remaining gray areas (to keep or remove) and the phenotype/trigger of the episode while reviewing the videos. Additionally, our FOGtool calculates the positive agreement, negative agreement, and prevalence index for the interrater agreement and displays them in an overview table.

The software (a stand‐alone executable, not requiring MATLAB installation) and the code are freely accessible on https://github.com/helenacockx/FOGtool and includes a clear instruction manual.

## Discussion

The hereby proposed guideline and open‐source FOGtool contributes to standardization of FOG assessment by describing a procedure to combine video annotations of two raters. Furthermore, we promote the use of positive agreement, negative agreement and prevalence index to report the interrater agreement instead of using the intraclass correlation coefficient or the Cohen's kappa. Application of the proposed procedure, or at least reporting of the two parameters (tolerance and correction) should improve transparence on video‐based FOG assessment, thereby helping to interpret shared datasets and compare study results over centers.

## Author Roles

1) Research project: A. Conception, B. Organization, C. Execution; 2) Statistical Analysis: A. Design, B. Execution, C. Review and Critique; 3) Manuscript: A. Writing of the first draft, B. Review and Critique.

H.C.: 1A, 1B, 1C, 3A.

E.K.: 1A, 1C, 3B.

M.T‐C.: 1A, 3B.

R.vW.: 1A, 3B.

J.N.: 1A, 3B.

## Disclosures

### Ethical Compliance Statement

The authors confirm that the approval of an institutional review board or patient consent was not required for this work. We confirm that we have read the Journal's position on issues involved in ethical publication and affirm that this work is consistent with those guidelines.

### Funding Sources and Conflicts of Interest

This work was supported by the Operational Program European Regional Development Fund (OP ERDF) of the European Union under the “PROMPT” project (PROJ‐00872) and the “Nederlandse Organisatie voor Wetenschappelijk Onderzoek–Toegepaste en Technische wetenschappen” (NWO‐TTW) Crossover program under the Innovative NeuroTechnology for Society (INTENSE) project. The authors declare that they have no competing interests concerning the research related to this manuscript.

### Financial Disclosures for the Previous 12 Months

H. Cockx and R. van Wezel report support from Operational Program European Regional Development Fund (OP ERDF) of the European Union and the “Nederlandse Organisatie voor Wetenschappelijk Onderzoek—Toegepaste en Technische wetenschappen” (NWO‐TTW) Crossover program. E. Klaver and M. Tjepkema‐Cloostermans report grants from the Michael J. Fox Foundation (Legacy ID 16457) outside the submitted work. Dr. Nonnekes reports grants from ZonMW (OffRoad grant, Veni grant), Michael J. Fox Foundation, Ipsen Pharmaceuticals, and Gossweiler Foundation outside the submitted work. He serves at the medical advisory board of Cue2Walk and Ceriter.

## Supporting information


**Supplementary Material**: Formulas for the reliability and agreement parameters.
