# Supplementary material for: The Gray Area of Freezing of Gait Annotation: A Guideline and Open‐Source Practical Tool
Source: Mov Disord Clin Pract. 2022 Sep 18;9(8):1099–104. doi: 10.1002/mdc3.13556 (PMC9631855; doi:10.1002/mdc3.13556)
Supplement: Supplementary file 1 — Supplementary Material: Formulas for the reliability and agreement parameters. [file MDC3-9-1099-s001.pdf]

Supplementary material

Supplementary table 1

Formulas for the reliability and agreement parameters

		rater 2	
		FOG	no FOG
rater 1	FOG	a	b
	no FOG	c	d

$$n = a + b + c + d \qquad (1)$$

Where *a* and *d* are respectively defined as the total duration of overlapping areas that are definitely considered as freezing (*a,* black areas), or no freezing (*d,* white areas); *b* and *c* are defined as the total duration of non-overlapping FOG episodes (grey areas); and *n* is defined as the total duration of gait tasks considered for FOG assessment.

Interrater reliability (15, 16)

Different formulas exist, depending on the context, see (18) or (20).

Cohen's kappa (14)

$$\kappa = \frac{p_o - p_c}{1 - p_c} \qquad (2)$$

Where κ is the Cohen's kappa, p_o the proportion of observed agreement, and p_c the proportion of agreement expected by by chance. p_o and p_c are respectively defined as:

$$p_o = \frac{(a + d)}{n} \qquad (3)$$

$$p_c = \frac{\frac{(a + c)(a + b)}{n} + \frac{(b + d)(c + d)}{n}}{n} \qquad (4)$$

Positive agreement (15)

$$positive\ agreement = \frac{2a}{n + (a - d)} \qquad (5)$$

Negative agreement (15)

$$negative\ agreement = \frac{2d}{n-(a-d)} \quad (6)$$

Prevalence index (14)

$$prevalence\ index = \frac{a-d}{n} \quad (7)$$

REFERENCES

The reference numbers of the main text were used.

14. Sim J, Wright CC**.** The Kappa Statistic in Reliability Studies: Use, Interpretation, and Sample Size Requirements. Physical Therapy. 2005;85(3):257-68.
15. Cicchetti DV, Feinstein AR**.** High agreement but low kappa: II. Resolving the paradoxes. J Clin Epidemiol. 1990;43(6):551-8.
16. Gisev N, Bell JS, Chen TF**.** Interrater agreement and interrater reliability: key concepts, approaches, and applications. Res Social Adm Pharm. 2013;9(3):330-8.
18. McGraw KO, Wong SP**.** Forming inferences about some intraclass correlation coefficients. Psychological Methods. 1996;1(1):30-46.
20. Shrout PEF, Josep L. Intraclass Correlations: Uses in Assissing Rater Reliability. Psychological Bulletin, 1979; 86.
